# Wood identification of *Cyclobalanopsis* (Endl.) Oerst based on microscopic features and CTGAN-enhanced explainable machine learning models

**DOI:** 10.3389/fpls.2023.1203836

**Published:** 2023-07-07

**Authors:** Weihui Zhan, Bowen Chen, Xiaolian Wu, Zhen Yang, Che Lin, Jinguo Lin, Xin Guan

**Affiliations:** ^1^ College of Materials Engineering, Fujian Agriculture and Forestry University, Fuzhou, Fujian, China; ^2^ College of Transportation and Civil Engineering, Fujian Agriculture and Forestry University, Fuzhou, Fujian, China; ^3^ National Forestry and Grassland Administration Key Laboratory of Plant Fiber Functional Materials, Fuzhou, Fujian, China

**Keywords:** *Cyclobalanopsis* (Endl.) Oerst, wood identification, machine learning, CTGAN, LIME

## Abstract

**Introduction:**

Accurate and fast identification of wood at the species level is critical for protecting and conserving tree species resources. The current identification methods are inefficient, costly, and complex

**Methods:**

A wood species identification model based on wood anatomy and using the *Cyclobalanopsis* genus wood cell geometric dataset was proposed. The model was enhanced by the CTGAN deep learning algorithm and used a simulated cell geometric feature dataset. The machine learning models BPNN and SVM were trained respectively for recognition of three *Cyclobalanopsis* species with simulated vessel cells and simulated wood fiber cells.

**Results:**

The SVM model and BPNN model achieved recognition accuracy of 96.4% and 99.6%, respectively, on the real dataset, using the CTGAN-generated vessel dataset. The BPNN model and SVM model achieved recognition accuracy of 75.5% and 77.9% on real dataset, respectively, using the CTGAN-generated wood fiber dataset.

**Discussion:**

The machine learning model trained based on the enhanced cell geometric feature data by CTGAN achieved good recognition of *Cyclobalanopsis*, with the SVM model having a higher prediction accuracy than BPNN. The machine learning models were interpreted based on LIME to explore how they identify tree species based on wood cell geometric features. This proposed model can be used for efficient and cost-effective identification of wood species in industrial applications.

## 1 Introduction

Wood classification is a fundamental and essential task in wood science and technology. It enables the identification of various wood species and ensures their sustainable utilization. Wood can be classified into different levels: kingdom, division, class, order, family, genus, and species (Wheeler and Baas, 1998; Martins et al., 2013). In wood classification, identifying wood at the ßpecies” level is often regarded as the most arduous task. ßpecies” is the primary classification unit that refers to individuals of the same species with the same morphological characteristics, chemical composition, and tissue structure (Wheeler et al., 1989; Mai et al., 2022). The classification of wood at the ßpecies” level demands an in-depth comprehension of the differences among individuals of the same species, necessitating researchers to possess high levels of expertise and skills. According to the recent report “State of the World’s Trees” by the Botanic Gardens Conservation International (BGCI), nearly 30% of tree species globally were in danger of extinction, with 27% under threat from the expanding wood trade. Consequently, wood regulation has become a significant challenge in safeguarding tree species. In this context, wood identification plays a crucial role. Therefore, research on wood classification at the ßpecies” level has tremendous importance in conserving forest resources and promoting the wood trade, thereby providing the wood industry with enhanced quality control and management (Gasson et al., 2010; Koch et al., 2015; Wiedenhoeft et al., 2019).

Wood identification is a complicated process. Experts use traditional wood identification techniques based on macroscopic and microscopic wood anatomy (Kuroda, 1987; Coday et al., 1997; Romagnolj et al., 2007). These techniques can identify tree species from raw wood, sawn wood, and finished products. Macroscopic identification serves as a supplemental reference to microscopic identification. The latter compares the morphological characteristics of tissues and cells in three sections of wood samples with accurately named wood specimens, enabling more precise identification (Jansen et al., 1998; Carlquist, 2013; Zhang et al., 2014). To attain accurate wood identification, experts and scholars have conducted research using quantitative identification methods for cell features. Experts use quantitative data to study patterns of cellular structural characteristic variation between tree species and subtle structural differences (Gasson et al., 2010). Nevertheless, manually identifying microscopic features is time-consuming, and some wood species have substantial inter-species variation. Personnel assigned with identification may encounter difficulty in mastering the variation rules of all wood species and struggle to discern nuanced structural differences between them without the help of a microscope. These factors add to the challenges of identifying wood at the species level, necessitating significant effort from researchers (Richter et al., 2004; Wheeler, 2011; Angyalossy et al., 2016).

In recent years, numerous methods have been developed and studied for wood identification, including molecular markers, spectral chemical analysis, stable isotopes, and other techniques (Ohyama et al., 2001; Grabner et al., 2009; Finkeldey et al., 2010; Kobayashi et al., 2019a; Sharma et al., 2020).While these methods have enabled identification at the ßpecies” level, significant investments in workforce and financial resources are required to build corresponding classification feature databases. However, advances in artificial intelligence (AI) technology have led to the emergence of new ideas to facilitate rapid and accurate identification at the ßpecies” level (Tou et al., 2007; Yuliastuti et al., 2013; Mohan et al., 2014). Machine learning forms the core of AI, withdeep learning constituting a large-scale machine learning approach often employing multilayer convolutional neural networks and deep, fully connected neural networks to construct models (Yadav et al., 2015a; Hwang and Sugiyama, 2021). These models rely on vast amounts of input data and significant computing power to gain a deeper understanding of knowledge. However, as the complexity of AI models continues to increase, the models themselves are becoming increasingly opaque, with input and output processing often complicated to comprehend (Sun et al., 2021). Within the field of wood identification, deep learning based on computer vision has been utilized for building models to classify wood species successfully (Hafemann et al., 2014). Researchers aim to capture the microscopic structural characteristics of wood by using a microscope, with image-based data being leveraged as input for the computer vision classification models. The characteristics of these models include complex calculations, large amounts of data, slow training, and relatively poor interpretability. To better facilitate rapid and accurate tree species recognition modeling, rigorous research in feature extraction, data preprocessing and enhancement, model selection, and evaluation is necessary.

The Fagaceae family comprises over 900 species distributed worldwide in Eurasia’s temperate and subtropical forests. In China, seven Fagaceae genera are identified: Castanopsis, Quercus, *Cyclobalanopsis*, Lithocarpus, Fagus, Castanea, and Trigonobalanus, with more than 300 species. The *Cyclobalanopsis* genus is the most prevalent, with approximately 80 species predominantly found in the Qinling Mountains and south of the Yangtze River. One Fagaceae species is listed under Appendix II of the Convention on International Trade in Endangered Species of Wild Fauna and Flora (CITES). Six species are listed as Class II National Key Protected Wild Plants in China. Developing a precise and prompt identification method at the level of “species” is crucial to safeguard and sustainably use tree species resources (Lions, 2011; Bergesen et al., 2018). Therefore, this research aims to create a high-quality species identification model based on wood samples, focusing on the *Cyclobalanopsis*genus of Fagaceae.

In wood identification, research in artificial intelligence models has two main directions: popular deep learning models based on image e data (Kwon et al., 2017; Ravindran et al., 2018) and machine learning models based on specific quantitative values (Sugiarto et al., 2017; He et al., 2019; Lens et al., 2020; Liu et al., 2022). While both are valid methodologies, the latter commonly uses traditional machine learning models with relatively simple structures, making them faster to train and easier to comprehend. Consequently, such models have significant potential for application within the field of wood identification. Regarding data types, there are four types of data in wood identification models: microscopic images, stereograms images, CT images, and macroscopic images (Hwang and Sugiyama, 2021). Traditional wood science suggests that microscopic images have the highest identification accuracy, with other images often used as auxiliary means of wood identification. Therefore, training a wood identification model based on microscopic images is the best choice. In the past decade, research on computer tree species recognition based on microscopic images has indicated that feature selection for microscopic image recognition models can generally be divided into image features and tabular numerical features (**Table 1**). Convolutional networks based on image features often extract convolutional features through convolutional layers, which are difficult to understand and confusing for wood science researchers. In contrast, machine learning models based on tabular numerical features are relatively easy to understand. However, the feature extraction method used in previous research was based on computer graphics rather than wood anatomy, which still needs to be more user-friendly for wood science researchers. As a result, we are approaching this problem from a wood anatomy perspective, and it is feasible to establish a wood identification model based on the geometric features of the wood anatomy dataset. Therefore, we extracted the geometric features of *Cyclobalanopsis* wood vessel cells and wood fiber cells as training features in this study. By comparing models trained on the geometric feature dataset of the two cell types, it could effectively study the specific impact of each cell type regarding the inter-species identification of *Cyclobalanopsis* wood.

**Table 1:** Research on CV-based microscopic image identification of wood in the last 10 years.

References	Model	Feature	Feature num	The best classification rate(%)
Ravindran and Wiedenhoeft (2020)	Deep learning	Convolution features		96.10%
Lens et al. (2020)	Deep learning	Convolution features		95.60%
Hwang et al. (2020)	Machine learning	SIFT	128	99.40%
Kobayashi et al. (2019b)	Machine learning	SIFT+Connected component labelling	17 + 17	95.30%
Rosa da Silva et al. (2017)	Machine learning	LPQ+LBP	256 + 4116	90.00%
Hwang et al. (2018)	Machine learning	SIFT	128	96.30%
Yadav et al. (2015b)	Machine learning	LBP	325	97.87%
Yadav et al. (2014)	Machine learning	Coiflet Discrete Wavelet Transform	48	92.20%
Hafemann et al. (2014)	Deep learning	Convolution features		97.32%
Martins et al. (2013)	Machine learning	Image Structural Features+GLCM+LBP	5 + 24 + 59	98.60%
Yadav et al. (2013)	Machine learning	GLCM	44	92.00%

However, due to the complexity of the internal structure of wood cells, collecting geometric feature data requires a substantial amount of time and labor (Von Arx et al., 2016; von Arx et al., 2021). Researchers must further explore data preprocessing and enhancement techniques. Using a deep learning network for feature modeling is a viable solution based on the similarity of geometric feature data within the same wood species. In a related study, Xu et al. (2019) proposed the conditional tabular generative adversarial network(CTGAN), which synthesized tabular dataset. CTGAN was compared with Bayesian and other deep learning methods on seven simulated and eight real dataset. Results revealed that CTGAN outperforms the alternative methods on most datasets, demonstrating a more extraordinary data generation ability that aligns closely with actual data distribution (Assefa et al., 2020; Bourou et al., 2021; Torfi et al., 2022). The robust data generation capabilities of CTGAN have earned the trust of many researchers. As a reliable generation model, CTGAN can help researchers generate data that would otherwise be costly. CTGAN has been applied in various fields, such as electroencephalogram, power system data generation, privacy medical data generation, mobile sensor data generation, and financial asset configuration data generation (Lee and Lee, 2021; Han et al., 2022; Fang et al., 2022; DeOliveira et al., 2022; Peña et al., 2023; Cifuentes et al., 2023). However, the application of generation models in the field of wood science is scarce, and the workload and cost of collecting data for training wood identification models or wood property prediction models are enormous. As a result, in this study, the CTGAN model was used to augment the geometric feature dataset of *Cyclobalanopsis* wood, effectively expanding the scale of the data and improving its diversity and reliability.

General wood identification models often face complex input data and network structure challenges. Typically, evaluations of these models often lack explanations of the models themselves, only providing accuracy on validation sets or confusion matrices as indicators of model quality (Ribeiro et al., 2016). While models perform well in wood identification, they may provide an overestimated recognition effect as real-world data differ significantly from simple validation sets. Therefore, model interpretability is necessary to provide insights into wood science and anatomy development. Expressly, model interpretability permits the analysis of the impact of different component factors on wood identification to create improved identification based on feature impact data (Vellido et al., 2012; Milli et al., 2019). Additionally, interpretable models provide essential explanations to non-data science professionals and the general public, establishing trust in the artificial intelligence model in wood science and the wood industry to enable people to make more informed decisions based on prior knowledge of wood science. For these reasons, evaluations of the model should also include scientific explanations of the model in addition to traditional measures of accuracy and confusion matrix. From the observation of **Table 1**, machine learning models have an advantage over deep learning models when explaining wood species identification models based on microscopic images. Deep learning models require images as input, yielding convolutional features that are challenging to explain. On the other hand, machine learning models can establish tabular feature datasets based on wood anatomy, with a more intuitive feature selection range, making it easier for wood science researchers to understand the model. As a preliminary study of wood identification model interpretability, this paper will use Local Interpretable Model-Agnostic Explanations (Ribeiro et al., 2016) on machine learning models to interpret the wood identification model, and a simple linear model will approximate the prediction field of interest. The weight coefficients of geometric features will explain how the model identifies the wood species (Mishra et al., 2017; Peltola, 2018).

This study utilized the CTGAN-enhanced simulated *Cyclobalanopsis* wood cell geometric feature dataset to establish two machine learning models (SVM, BPNN) for wood identification. The models were tested and evaluated on an actual *Cyclobalanopsis* wood cell geometric feature dataset, and the interpretability of the wood recognition model was demonstrated using Local Interpretable Model-Agnostic Explanations (LIME). Thus, this study provides the following contributions:

• It proposed a novel wood recognition model based on microscopic images that leveraged wood anatomy principles and cell geometrical feature data to enhance accuracy. Compared with previous wood identification models based on microscopic images, the current work differs in that it extracts a small amount of anatomical feature data and combines it with prior knowledge of wood anatomy instead of extracting complex convolution features and local point-line-face features. The proposed model integrates more with previous knowledge of wood anatomy through artificial feature extraction and model training. Based on artificial neural network analysis, the study found that cell geometrical features, particularly those determined by vessels, significantly impacted wood identification accuracy more than features based on wood fibers.
• The CTGAN model was utilized based on deep learning to augment the wood’s quantitative cell geometrical feature data, representing the first time such an approach has been used. The feasibility of this method was evaluated using a real wood cellgeometry dataset. CTGAN model dramatically reduces the cost of manually collecting anatomical features, enabling wood scientists to train their species identification models relatively quickly based on the wood anatomical features.
• Furthermore, interpretation of wood identification models was researched by examining the impact of various wood cell geometrical features of *Cyclobalanopsis* on models. This study presented the first of its kind. The findings of our study offered a quantitative and qualitative understanding of interpretable models for wood anatomy and also provided valuable insights for improving feature engineering of future artificial intelligence-based wood identification systems, thereby advancing the field of wood science.

## 2 Experiments and methods

### 2.1 Sample collection

Sample trees were collected from the middle subtropical region of Fujian province, China. The discs near ground with 5.0 cm thickness and logs above ground 1.3 m (breast height) with 2.0 m length were got from the straight and healthy average stem in the stand. Then discs and logs as wood samples were placed in a shaded and well-ventilated indoor environment until air drying. The general information of the tree samples was shown in **Table 2**.

**Table 2:** Tree species and collecting locations.

Latin name	Tree Age/year	Genus Name	Collecting locations	Material colour	Scents
*Cyclobalanopsis gilva (Blume) Oerst*	35	*Cyclobalanopsis (Endl.) Oerst*	Jian’ou, Fujian	Sapwood is yellow-brown and the heartwood is red-brown to shades of red-brown	No distinct odor
*Cyclobalanopsis chungii (Metc.) Y. C. Hsu et H. W. Jen ex Q. F. Zheng*	33	*Cyclobalanopsis (Endl.) Oerst*	Minqing, Fujian	heartwood is red-brown and the sapwood is yellow-brown	No distinct odor
Cyclobalanopsis glauca(Thunb.) Oerst.	33	*Cyclobalanopsis (Endl.) Oerst*	Pingnan, Fujian	Heartwood color was similar to sapwood color with yellow-brown	No distinct odor

**Figure 1:**
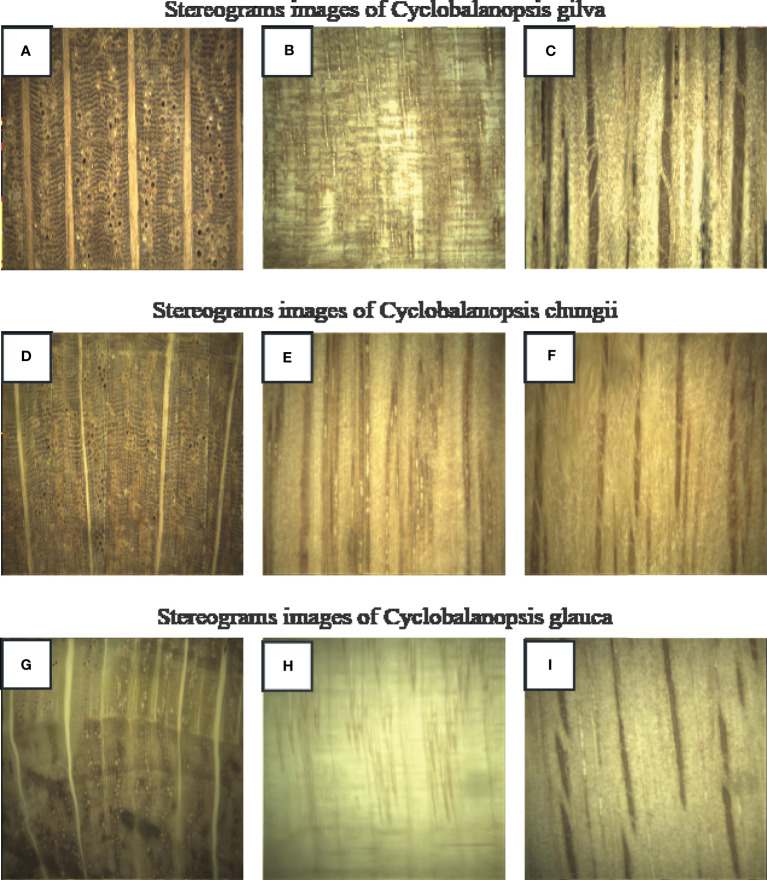
Images of the three species of Cyclobalanopsis under a stereomicroscope. **(A, D, G)** is cross section; **(B, E, H)** is radial section; **(C, F, I)** is tangential section.

Using a continuous zoom stereomicroscope (MZS0745, Guilin Mete Optical Instrument Co., Ltd.), stereograms of three types of Cyclobalanopsis wood were captured at 10X magnification, as shown in **Figure 1**. The macroscopic features of Cyclobalanopsis gilva wood were shown in **Figures 1A–C**. Heartwood color was distinct from sapwood color, with the sapwood being yellow-brown and the heartwood being red-brown to shades of red-brown. There was no special odor. The growth ring boundaries were indistinct with a uniform width. The macroscopic features of Cyclobalanopsis chungii wood were shown in **Figures 1D–F**. Heartwood color was distinct from sapwood color, with the heartwood being red-brown and the sapwood being yellow-brown. There was no special odor. The growth ring boundaries were indistinct, which width was not uniform with an average value of 3.03mm. The macroscopic features of Cyclobalanopsis glauca wood were shown in **Figures 1G–I**. Heartwood color was similar to sapwood color with yellow-brown. There was no special odor. The growth ring boundaries were distinct with a uniform width.

### 2.2 Wood microstructure production

The air-dried wood was intercepted into a standard three-cut small test block (10mm×10mm×10mm), and to ensure that the cross-section had at least one complete annual ring. The blocks were boiled in distilled water for 8h, and then soaked in distilled water at 26°C for 12h until the specimen were soft. After that, using a sliding microtome (REM-710, Daiwa Optical Machinery Co., Ltd., Japan), sections with a thickness between 10 and 20 were prepared from the wood specimens. All sections were stained with 1% safranin for 3.5 h and subsequently dehydrated using a series of alcohol concentrations (30%, 50%, 75%, 85%, 95%, 100%) for 5 min each. The fully dehydrated sections were placed in 100% xylene for clear treatment for 10 min and repeated once. Permanent slides were produced by sealing the slices with neutral resin. Slices were placed under a biological digital microscope (Leica DM2500, Leica Microsystems, Germany) to observe the microscopic characters of the wood. The Leica Application Suite software was used to extract data on microscopic features such as tangential diameter of vessel and vessel lumina, wall thickness of vessel, area of vessel and vessel lumina, perimeter of vessel and vessel lumina, substantive rate of vessel and fiber, area of fiber and fiber lumina.

### 2.3 Model building

#### 2.3.1 Conditional tabular generative adversarial network

CTGAN is a deep learning model that employs conditional generative adversarial networks (Mirza and Osindero, 2014) to model the probability distribution of tabular data rows and synthesize data with features closely related to the input data. The model accomplishes this through a game between two neural networks: the generator and the discriminator. The generator learns the probability distribution of accurate data and generates high-quality synthetic data. The discriminator constantly judges the generated data and gives feedback to optimize the weight of the neural network. The generator network comprises three fully connected layers that employ batch normalization and LeakyReLU activation functions (Maas et al., 2013). In comparison, the discriminator network has three fully connected layers that use batch normalization, LeakyReLU activation functions, and the final layer with the sigmoid activation function. During the training phase, the generator and discriminator work together to ensure that the synthetic data generated by the generator are indistinguishable from the actual data. The input to the model consists of a noise vector and a conditional vector, while the output is the synthesized data.

The objective function of the Generative Adversarial Network is shown in Equation 1.


(1) $$\underset{G}{\min}\underset{D}{\max}V\left(D,G\right)=E_{x∼P_{\text{data }}\left(X\right)}\left[\log\;(D\left(x\right)\right)]+E_{x∼P_{z}\left(z\right)}\left[\log\;(1-D\left(G\left(z\right)\right)\right)]$$

**Figure 2:**
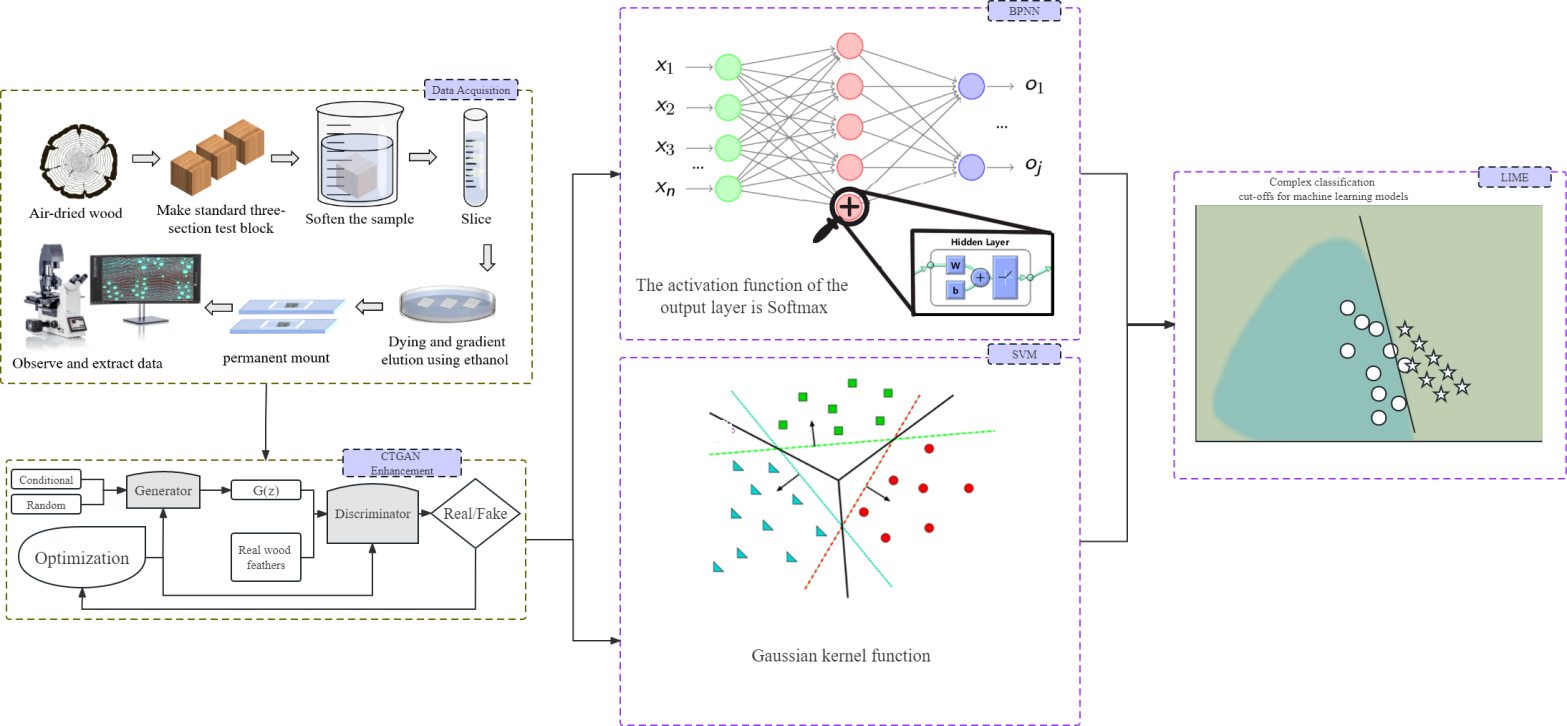
Machine learning model workflow based on CTGAN, wood cell geometry features figures and LIME.

In the equation, G represents the generator, and D represents the discriminator. The generator and discriminator are trained iteratively until the discriminator cannot distinguish the authenticity of the generated data. Once this occurs, the Generative Adversarial Network (GAN) model is optimized (224 Courville and Bengio, 2014). **Figure 2** shows the machine learning model based on CTGAN established in this study. The generator takes the *Cyclobalanopsis* wood cell geometrical feature data as a conditional input, adds random noise, and sends it to the discriminator for evaluation. The feedback from the discriminator is then used to adjust the network weights of the generator, which gradually optimizes the generated data features to mirror that of the actual dataset. CTGAN can significantly reduce the cost of collecting cell features, enabling this experiment to train a robust and reliable model with only a tiny amount of geometrical features collected from wood cells.

#### 2.3.2 Backward propagation neural network

Artificial Neural Network (ANN) is a commonly used machine learning technique in various fields, including classification, prediction, optimization, and other tasks (Ingre and Yadav, 2015). The Back Propagation-Artificial Neural Network (BPNN) algorithm is widely used for quantitatively modeling numerical features. It is a simple multi-layer neural network with multiple layers of neurons trained using the backpropagation algorithm (Rumelhart et al., 1986). The BPNN’s basic structure includes inputs, outputs, and multiple hidden layers of neurons. Each neuron’s output is connected to the neurons in the previous layer, forming a multi-layer feedforward neural network structure (Agatonovic-Kustrin and Beresford, 2000).

As illustrated in **Figure 2**, the BP artificial neural network comprises three layers of neurons: the input layer, hidden layer, and output layer. The hidden layer consists of two layers and 20 nodes. The *Cyclobalanopsis* wood cell geometrical feature data was collected through software, enhanced by CTGAN, and standardized before passing through the input layer to the second layer, i.e., the hidden layer, where weight transfer occurs. In the hidden layer, each neuron was activated by weight W, threshold b, and the Relu activation function and transmitted to the output layer. The output layer generated the predicted value of the neural network and compared it with the expected value. Any errors detected were back propagated from the output layer, and the weights and thresholds were adjusted. The repeated training and adjustment process continued until the output error reached an acceptable level. The specific model calculation was shown below.

The model was propagated in the forward direction to generate the predicted output ${\hat{y}}_{i}$ . The cross-entropy loss function was first used to evaluate the error between the predicted value ${\hat{y}}_{i}$ and true value y_i_. The cross-entropy loss function determined the better prediction model by the maximum likelihood estimate of the correct prediction for each set of data. The smaller cross-entropy could get the lower model prediction error (2).


(2) $$loss=-\sum_{\text{i}=1}^{\text{n}}{y_{i}\log\hat{y}}_{i}$$

In the first backpropagation step, the gradient value $\frac{\partial \;loss\;}{\partial w_{o}}$ of the backpropagation process with respect to the output layer weight ${\text{w}}_{\text{o}}$ was obtained by taking the derivative of error with respect to the weight ${\text{w}}_{\text{o}}$ . y represented the true value and $\hat{y}$ represented the predicted value which was operated by the Softmax activation function in the output layer(3).


(3) $$\frac{\partial loss}{\partial w_{o}}=\frac{\partial loss}{\partial y}\frac{\partial \hat{y}}{\partial w_{o}}$$

The input values of the Softmax activation function were a set of vectors of tree species prediction scores, and the output consisted of a prediction vector of identification probabilities for the corresponding tree species(4).


(4) $$f_{j}\left(z\right)=\frac{e^{z_{j}}}{\sum_{k}e^{z_{k}}}$$

In the second backpropagation step, the gradient value $\frac{\partial \;loss\;}{\partial w_{h}}$ in the backpropagation process with respect to the hidden layer weight ${\text{w}}_{\text{h}}$ was obtained by taking the derivative of the error with respect to the weight ${\text{w}}_{\text{h}}$ . h represented the Sigmoid activation function in the hidden layer(5).


(5) $$\frac{\partial loss}{\partial w_{h}}=\frac{\partial loss}{\partial y}\frac{\partial \hat{y}}{\partial h}\frac{\partial h}{\partial w_{h}}$$

The activation function h for the j node in the hidden layer could be formulated as equation 6:


(6) $$\text{h}=f\left(x_{j}\right)=\frac{1}{1+exp\;\left(-x_{j}\right)}$$

4. Finally, the weights of output and hidden layers were updated by gradient descent(7).


(7) $$w_{\text{update }}=w_{\text{old }}-\eta \frac{\partial \;loss\;}{\partial w_{old\;}}$$

Where $w_{\text{update }}$ represented the weights of hidden and output layers after updating, $w_{\text{old }}$ represented the weights of hidden and output layers before updating, η represented the model learning rate, and $\frac{\partial xtloss}{\partial w_{old\;}}$ was the gradient of corresponding weights.

5. The updated weights were substituted into the forward propagation for a new prediction, and the error was made to reach an acceptable range by repeating the above steps continuously. Finally, the predicted value was output.

The standard BP neural network algorithm’s gradient descent algorithm (Ruder, 2016) adjusts the network weights and thresholds along the negative gradient direction of a network error. This eventually causes the error to reach a minimum value.

#### 2.3.3 Support vector machine

Support Vector Machine (SVM) is a machine learning algorithm based on statistical VC dimension theory and structural risk minimization (Joachims, 1998). Initially applied to binary classification, it can also solve multi-classification problems and effectively address small sample, non-linear, high-dimensional, and local minimum problems. The core of SVM lies in finding the maximum hyperplane in high-dimensional space to separate sample data, making the classification reach the maximum interval, given a point x on the hyperplane, and ω as a vector perpendicular to the hyperplane as displacement interval, and b as the shift interval, the maximum interval can be represented as equation 8. If $\frac{2}{\left\|\omega \right\|}$ is maximized, it is equivalent to minimizing $\|\omega {\|}^{2}$ . Equation 8 can be transformed into equation 9, the basic mathematical model of SVM.


(8) $$\{\begin{array}{c} \underset{\omega ,\text{b}}{\max}\frac{2}{\left\|\omega \right\|}\\ y_{i}\left(\omega \cdot x_{i}\pm b\right)\geq 1,i=1,2,\cdots ,m \end{array}$$

(9) $$\{\begin{array}{c} \underset{\omega ,b}{\min}\frac{1}{2}\|\omega {\|}^{2}\\ y_{\text{i}}\left(\omega \cdot {\text{x}}_{\text{i}}\pm \text{b}\right)\geq 1,\text{i}=1,2,\cdots ,\text{m} \end{array}$$

(10) $$y=\sum_{\text{i}=0}^{\text{m}}\omega_{i}^{\text{T}}\cdot {\text{x}}_{\text{i}}\pm \text{b}$$

In this research, the SVM classifier would be based on the Gaussian kernel function with a kernel scale of 3. The computation process of the SVM classifier was as follows: firstly, the geometric feature dataset of *Cyclobalanopsis* wood cells would be passed to the initial calculation module to obtain m-dimensional coefficients $\|\omega {\|}^{T}$ and a threshold value b . Then, equation 10 would multiply and sum the *Cyclobalanopsis* species wood cell geometric feature values x corresponding to each block with $\|\omega {\|}^{T}$ . Finally, by comparing the relationship between the classification calculation result and the threshold value b , the classification result of *Cyclobalanopsis* species wood cell geometric feature data could be obtained (Chang et al., 2005; Friedrichs and Igel, 2005; Xiao et al., 2014).

### 2.4 Model evaluation

In machine learning, a model’s classification capability is a crucial performance metric for evaluation purposes (McAvaney et al., 2001). During the process of evaluating model performance, various performance indicators are applied to obtain a comprehensive understanding of the model’s performance. Generally, accuracy and confusion matrix are the two most commonly used evaluation indicators.

Accuracy, represented by formula 11, is defined as the proportion of samples correctly classified to the total number of samples. TP denotes the count of correctly identified positive samples, and TN represents the count of correctly identified negative samples by the classifier(11).


(11) $$ACC=\frac{TP+TN}{\;TOTAL\;}$$

Despite being a widely used evaluation metric, accuracy needs to be improved for assessing the performance of classifiers in multi-class classification problems. Hence, the confusion matrix is a more suitable evaluation metric for comprehensively evaluating the model performance. The confusion matrix tabulates the misclassification frequency of each tree type by comparing the predicted and actual categories (Gauch et al., 2003; Raschka, 2018). The confusion matrix illustrates the predicted and true categories in columns and rows. Consequently, the matrix enables the identification of misclassified classes and their respective frequencies for each tree species.

### 2.5 Model interpretability

Interpretability refers to the degree to which humans can comprehend artificial intelligence algorithms, often called “black boxes,” since their knowledge representation is often counterintuitive, making it complicated to understand their behavior. Interpretability techniques facilitate revealing the rationale behind predictions generated by black box machine learning models(Zhang and Zhu, 2018; Poursabzi-Sangdeh et al., 2021) By identifying how features affect, or do not affect, predictions, interpretability techniques assess whether the model utilizes appropriate professional knowledge, thereby detecting any biases that might arise during training.

Machine learning methods are substantially employed in identifying wood species. However, these models’ complexity requires interpretable methods to unveil their decision-making procedures. This study suggested that LIME explain the model to address this issue. LIME is a technique for interpreting models, initially proposed in 2016 by Marco Ribeiro and colleagues, that assists in explaining the decision-making process of complex black-box models. LIME can enlighten us about the weight of variables, their contribution to specific predictions, or similar factors. In studies on identifying tree species based on wood geometry parameters, LIME can reveal the identification procedure of machine learning models based on distinct input cell features. Specifically, the LIME technique utilizes a local linear model to approximate a model’s prediction on specific data. This method is simpler to comprehend since it only focuses on specific data on local linear structures (Swirszcz et al., 2009; Lozano et al., 2011; Ribeiro et al., 2016). As shown in **Figure 2**, machine learning models often have very complex and convoluted boundaries that are difficult to understand. Using the LIME technique, a simple linear model is locally fitted to the data for the boundary, and the interpretability of the model is achieved through the weight coefficients of the linear model.

The parameters of the local model in the LIME technique are calculated by the following formula(12).


(12) $$\phi \left(x\right)=\underset{g\in G}{\text{argmin}}L\left(f,g,\pi_{x}\right)+\text{Ω}\left(g\right)$$

Among them, f is the original model, G is the hypothesis space of the locally interpretable model, $\pi_{x}$ is the distribution of data points similar to instance x , L is the loss function, and Ω(g) is the regularization term. By minimizing this formula, the optimal local model ϕ can be obtained, thus explaining the output of the original model on a specific instance.

Using the LIME technique, the explanation results of specific instances could be obtained, including the weight of each feature, the contribution to the prediction result, and so on. For the BP neural network model and SVM support vector machine based on wood geometry features for tree species identification, the predictions for each wood species based on different models and cell data would be explained by LIME. These explanation results could help us better understand the working mode and importance of the features of the model, thus improving the interpretability and reliability of the model.

## 3 Results and analysis

### 3.1 Microstructure of three species of wood

**Figure 3:**
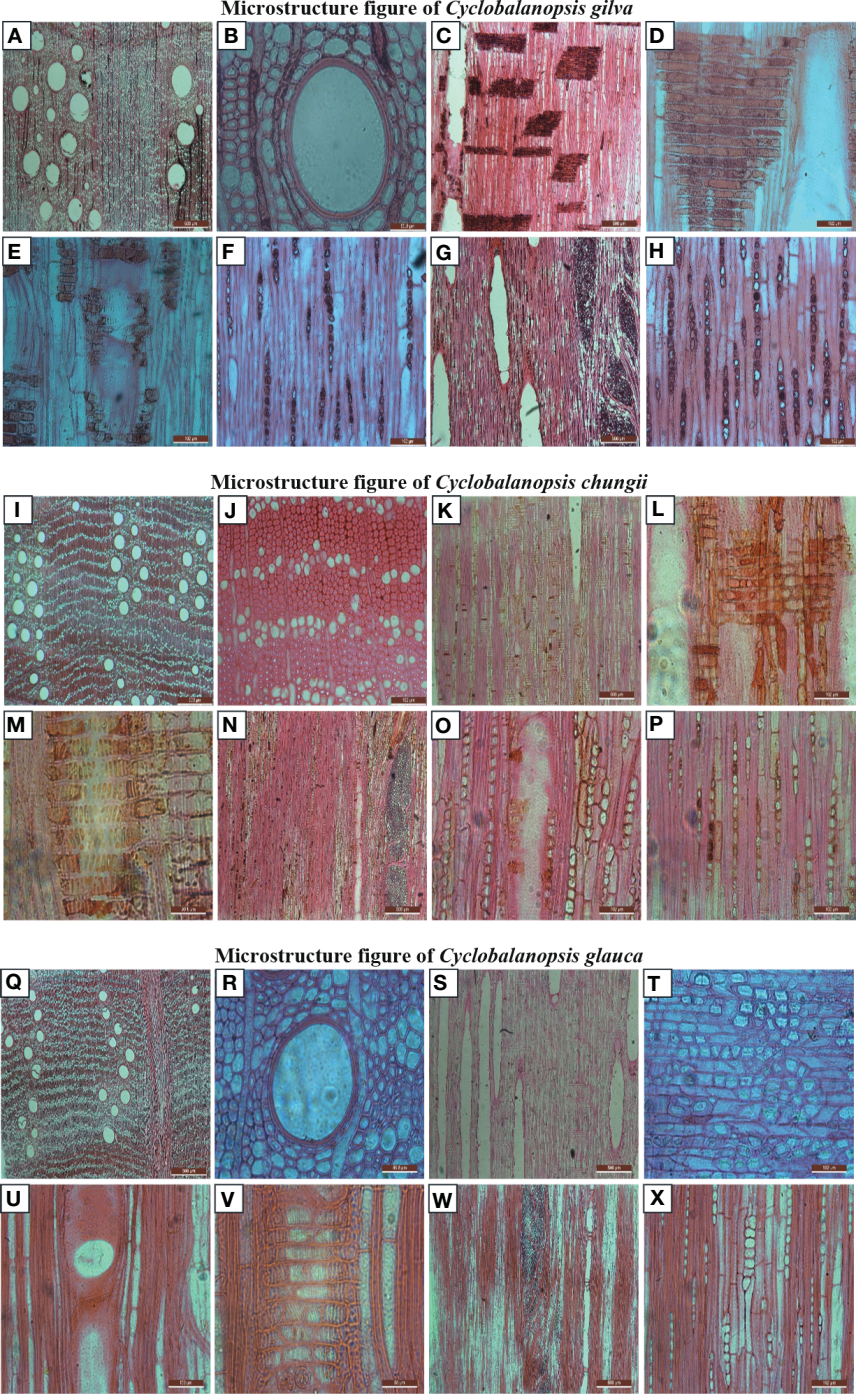
Microstructure Images of the three species of *Cyclobalanopsis* under a biological digital microscope. **(A–H)** is *Cyclobalanopsis gliva*; **(I–P)** is *Cyclobalanopsis chungii*; **(Q–X)** is *Cyclobalanopsis glauca*.

Vessels in cross section of *Cyclobalanopsis gilva* were solitary in radial pattern (**Figures 3A, B**). The tangential diameter of vessel lumina was in the range 74.19 through 314.56 with a mean value of 198.20. Vessels/mm2 was 3.12. Perforation plates were simple (**Figures 3C, G**). Helical thickenings were unpresented. Tyloses presented in some vessel lumina. Intervessel pits were alternate with the shape of rounded or angular (**Figures 3D, E**). Vessel-ray pits were with much reduced borders to apparently simple: pits vertical mostly (**Figure 3E**). Vasicentric tracheids presented. Axial parenchyma presented diffuse-in-aggregates numerously, in narrow bands or lines up to six cells wide commonly and diffuse rarely (**Figure 3A**). Wood rays were exclusively uniseriate with an average number of 10.6/mm. Rays were homocellular with all ray cells procumbent. Two types of wood rays presented: (1) uniseriate and 2-seriate rays with a height of 2 22 ray cells (**Figures 3F, H**); (2) aggregate rays with a breadth >11 ray cells (**Figure 3G**).

Vessels in cross section of *Cyclobalanopsis chungii* were solitary in a radial or diagonal pattern (**Figure 3I**). The tangential diameter of vessel lumina was in the range 39.86 through 202.77 with an average value of 139.27. Vessels/mm2 was 3.91. Perforation plates were simple. Helical thickenings were unpresented. Tyloses presented in some vessel lumina. Intervessel pits were alternate with the shape of rounded or oval (**Figure 3O**). Vessel-ray pits were with much reduced borders to apparently simple: pits rounded mostly (**Figures 3L, M**). Vasicentric tracheids presented. Fibers with simple pits were infrequent (**Figure 3P**). Axial parenchyma presented in narrow bands of 1 3 cells wide (**Figure 3I**). Wood rays were homocellular with all ray cells procumbent. Two types of wood rays presented: (1) uniseriate and 2-seriate rays with a height of 3 21 ray cells (**Figures 3O, P**); (2) aggregate rays with a breadth >11 ray cells (**Figure 3N**).

Vessels in cross section of *Cyclobalanopsis glauca* were solitary in a radial or dendritic pattern (**Figures 3Q, R**). The tangential diameter of vessel lumina was in the range 44.33 through 184.50 with an average value of 116.62. Vessels/mm2 was 3.4. Perforation plates were simple (**Figures 3S, U**). Helical thickenings were unpresented. Tyloses presented in some vessel lumina. Intervessel pits were alternate with the shape of rounded or oval. Vessel-ray pits were with much reduced borders to apparently simple: pits rounded mostly. Vasicentric tracheids presented. Axial parenchyma presented in narrow bands of 1 5 cells wide (**Figure 3Q**). A majority of crystals presented in aggregate rays (**Figure 3T**). Wood rays were exclusively uniseriate with an average number of 7.38/mm. Wood rays were homocellular with all ray cells procumbent. Two types of wood rays presented: (1) uniseriate and 2-seriate rays with a height of 3 20 ray cells (**Figure 3X**); (2) aggregate rays with a breadth >11 ray cells (**Figure 3W**).

### 3.2 Microstructural data of three species of wood

54 sets of microscopic features data from vessel elements and 96 sets of microscopic features data from wood fibers were collected for *Cyclobalanopsis gilva*, while 60 sets of data from vessel elements and 106 sets of data from wood fibers were gathered for *Cyclobalanopsis chungii*. For *Cyclobalanopsis glauca*, 49 sets of data from vessel elements and 92 sets of data from wood fibers were acquired (**Table 3**).

**Table 3:** Extracted data of microscopic features from three species.

	Microscopic features	Cyclobalanopsis gilva			Cyclobalanopsis chungii			Cyclobalanopsis glauca		
		Mean value	standard deviation	Coefficient of variation	Mean value	standard deviation	Coefficient of variation	Mean value	standard deviation	Coefficient of variation
Vessel elements	Wall thickness/µm	12.136	2.998	0.143	5.720	1.268	0.222	4.129	1.284	0.311
	Tangential diameter/µm	198.199	59.125	0.298	139.272	35.051	0.252	113.654	37.029	0.326
	Tangential diameter of lumina/µm	186.024	58.061	0.312	127.832	33.894	0.265	105.436	35.309	0.335
	ratio of wall to lumina	0.071	0.025	0.348	0.097	0.041	0.422	0.087	0.042	0.490
	Area/µm^2^	38603	21929	0.568	18529	8148	0.440	12481	6041	0.484
	Area of lumina/µm^2^	34613	20380	0.589	15894	7240	0.456	10848	5444.	0.502
	Substantial rate	0.118	0.040	0.340	0.156	0.046	0.293	0.138	0.041	0.297
	Circumference/µm	668.663	211.527	0.316	471.070	117.040	0.248	377.391	119.029	0.315
	Circumference of lumina/µm	630.738	207.618	0.329	435.361	113.410	0.260	351.415	112.736	0.321
Wood fibers	Area/µm^2^	206.634	53.536	0.259	143.274	41.509	0.290	106.891	40.613	0.380
	Area of lumina/µm^2^	89.580	39.339	0.439	18.198	12.782	0.702	21.006	12.838	0.611
	Substantial rate	0.568	0.143	0.251	0.919	0.036	0.039	0.805	0.080	0.100

In the genus *Cyclobalanopsis*, the anatomy of vessel cells is highly variable and can aid in species differentiation. As shown in **Table 3**, we compared the vessel elements and wood fiber characteristics of three *Cyclobalanopsis*species *: Cyclobalanopsis gilva*,*Cyclobalanopsis chungii*, and *Cyclobalanopsis glauca*.

The thickness of the vessel wall was significantly greater in *Cyclobalanopsis gilva* compared to the other two species. The tangential diameter and area of the vessel were also significantly more prominent in *Cyclobalanopsis gilva*. These features and the vessel’s circumference could be used to distinguish *Cyclobalanopsis gilva* from the other two species. The luminal size of *Cyclobalanopsis gilva* vessels was also more extensive, but this difference was not as significant as the other features. The ratio of wall to lumina was higher in *Cyclobalanopsis chungii* than in the other two species. While this feature is less significant than the vessel diameter, it could still contribute to species identification. The substantial rate of the vessels, which measures their compactness, was significantly higher in *Cyclobalanopsis chungii*. This feature alone may not be sufficient to differentiate the species, but it may contribute to the overall analysis.

The area of the wood fibers was significantly larger in *Cyclobalanopsis gilva* —however, more than this feature is required to differentiate among the species. The size of the fiber lumina was larger in *Cyclobalanopsis chungii*, but this difference was not as significant as in the vessel lumina. The substantial rate of the fibers was significantly higher in *Cyclobalanopsis chungii*, which could be combined with other features to differentiate species.

In conclusion, the vessel elements and wood fiber characteristics of *Cyclobalanopsis* spp. can be helpful in species differentiation. The significant differences observed in vessel wall thickness, tangential diameter, area, circumference, and wood fiber substantial rate can aid in identifying *Cyclobalanopsis gilva*, *Cyclobalanopsis chungii*, and *Cyclobalanopsis glauca*. In the following work, the CTGAN model based on the anatomical characteristics of *Cyclobalanopsis* wood was trained to produce reliable simulated anatomical feature data for training a tree species identification model. Finally, the LIME model’s explanation technique was used, compared, and discussed with the prior knowledge of the *Cyclobalanopsis* identification learned above.

### 3.3 Dataset construction and enhancement

**Figure 4:**
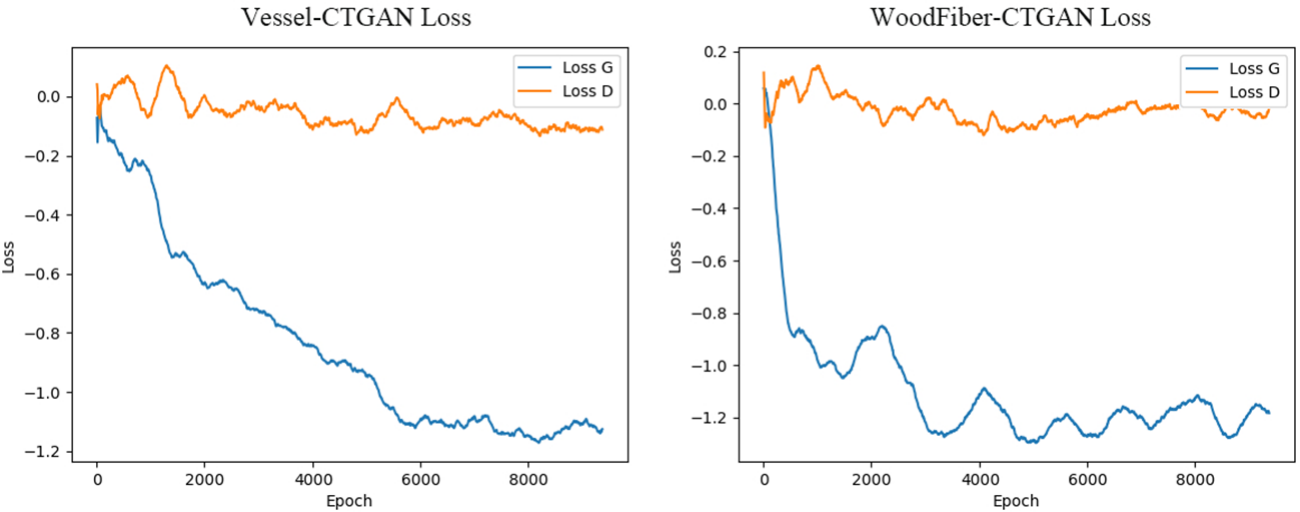
Variation in loss values for generators and discriminators.

Based on the geometric characteristics of real *Cyclobalanopsis* wood cells, we customized a *Cyclobalanopsis* cell simulation geometric data generation model using the CTGAN model through the application of Python’s SDV library, and the model was reliably evaluated by Python’s SDMetrics library. We used 163 sets of vessel cell data and 294 sets of wood fiber cell data to train the cell simulation geometric data generation model. Due to the relatively small amount of data in the training set, we used a relatively small batch size and a very long training period to improve the model’s generalization and feature extraction abilities. In order to avoid the problem of model overfitting caused by small batch sizes and long training periods, we used relatively small neural network dimensions and relatively small learning rates. We used the Adam optimizer for optimization. After multiple rounds of parameter adjustment, our final model training parameters are shown in **Table 4**, and the model training loss is shown in **Figure 4**. The generator loss of the Vessel-CTGAN model gradually stabilizes after 6000 rounds, and the discriminator loss also slowly converges. Meanwhile, the WoodFiber-CTGAN model’s generator loss stabilized at around -1.2 after 3000 rounds, and the discriminator also showed a stable trend, thus completing the model’s training.

**Table 4:** Model hyperparameters configuration.

Model	epochs	batch size	discriminator dim	discriminator decay	discriminator lr	discriminator steps	Embedding_dim	generator decay	generator dim	generator lr	pac	Optimizers
**Vessel-CTGAN**	10000	30	(256, 256)	1e-6	1e-4	1	128	1e-6	(256,256)	1e-4	10	Adam
**WoodFiber-CTGAN**	10000	20	(256, 256)	1e-6	1e-4	1	128	1e-6	(256,256)	1e-4	10	Adam
**Model**	**epochs**	**number of fully-connected layers**	**first layer size**	**second layer size**	**activation function**	**standardize**						
**BPNN**	1000	2	10	10	relu	true						
**Model**	**epochs**	**kernel**	**nuclear constraint**	**kernel scale**	**multi-class approach**	**standardize**						
**SVM**	1000	gaussian	1	12	One-to-one	true						

Synthetic data was generated using the CTGAN model based on a real geometric feature dataset of *Cyclobalanopsis*. The model learns from the *Cyclobalanopsis* wood cell geometrical feature dataset to generate synthetic data corresponding to actual *Cyclobalanopsis* wood geometric features’ statistical regularities. This expansion of the dataset was one of the benefits of this approach. The training dataset for wood geometrical features consisted of 163 groups of tracheal geometric data from *Cyclobalanopsis gilva*, *Cyclobalanopsis chungii*, and *Cyclobalanopsis glauca*, as well as 294 groups of wood fiber geometric data, trained for 5000 rounds.

In addition, the KS test was used to compare the differences in probability distribution between actual wood geometric feature data and generated wood geometric feature data. The KS test could be used to compare the difference between two probability distributions, and its core was to calculate the empirical cumulative distribution function (ECDF) of the two distributions. The KS statistic D represented the maximum difference between the two ECDFs. The larger the D value, the more significant the difference between the two distributions. The Ks complement was a supplementary index of the KS test, and its value equalled 1-D. Therefore, the larger the D value, the smaller the Ks complement value. The range of Ks complement was between 0 and 1. When the Ks complement value was closer to 1, it indicated that the two probability distributions were more similar; conversely, when the Ks complement value was closer to 0, it indicated that the two probability distributions were less similar. When Cifuentes et al. (2023) studied optimizing asset allocation data generation using CTGAN, the Ks complement indicator reached 0.87. Alqarni and El-Alfy (2022) studied generating network traffic intrusion detection data, and the Ks complement score ranged from 0.77 to 0.82. Peng et al. (2021) studied on generating credit rating data, the Ks complement indicator reached 0.88.

**Figure 5:**
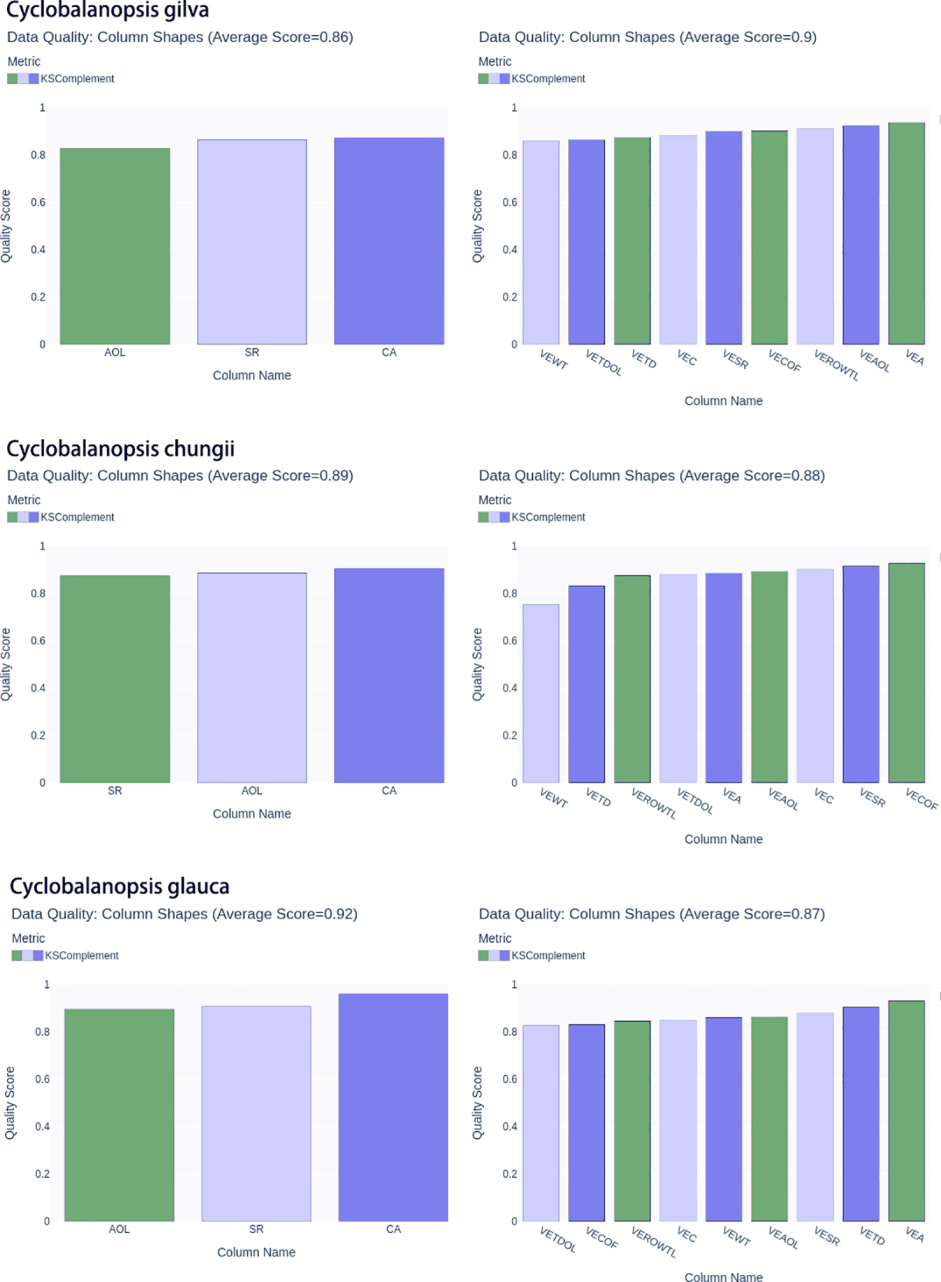
The KS complement metrics of the real and synthetic *Cyclobalanopsis* wood cell geometry datasets are presented, with the following letter symbols representing: vessel’s wall thickness (VEWT), vessel’s tangential diameter (VETD), vessel’s tangential diameter of lumina (VETDOL), vessel’s ratio of wall to lumina (VEROWTL), vessel’s area (VEA), vessel’s area of lumina (VEAOL), vessel’s substantial rate (VESR), vessel’s circumference (VEC), vessel’s circumference of lumina(VECOF),fiber’s area (CA), fiber’s substantial rate(SR), and fiber’s area of lumina (AOL).

In order to ensure accuracy and richness in data sampling, we divided the original real cell data into ten equal parts for each tree species. Then we carried out a single sampling of each of the equal parts to generate simulated data. For each equal part, we generated 100 simulation cell data samples, resulting in 1000 simulation cell data samples per tree species. The new data generated by CTGAN achieved good indicators on Ks complement (**Figure 5**). The average Ks complement of the vessel reached0.9, 0.88 and 0.87 corresponded to *Cyclobalanopsis gilva*, *Cyclobalanopsis chungii* and *Cyclobalanopsis glauca* in turn. The average Ks complement of the wood fibers reached 0.86, 0.89 and 0.92 corresponded to *Cyclobalanopsis gilva*, *Cyclobalanopsis chungii* and *Cyclobalanopsis glauca* in turn. Therefore, using the CTGAN model, 1000 groups of geometric data each for vessels and wood fibers were obtained, which could simulate the numerical distribution of natural wood geometric features of *Cyclobalanopsis gilva*, *Cyclobalanopsis chungii*, and *Cyclobalanopsis glauca*, respectively. SVM and BP neural networks would be trained based on 3000 groups of geometric feature data each for *Cyclobalanopsis*vessels and wood fibers.

### 3.4 Wood identification results

Using MATLAB, based on the hyperparameters configuration in **Table 4**, machine learning algorithm models were established to identify three species of *Cyclobalanopsis* by vessel and wood fiber geometry data through BP neural network and SVM support vector machine methods. The training of machine learning models is based on CTGAN-enhanced synthetic cell datasets. In order to verify the recognition effect of the model trained on CTGAN-enhanced data on real cell images, two machine learning models based on different synthetic cells were tested on both synthetic cell test sets and real cell datasets. The recognition results are shown in the **Table 5**, and the model trained on synthetic data can also achieve excellent recognition on real microscope features. The machine learning model based on Vessel-CTGAN-BPNN achieved high recognition rates of 99.2% in the test set and 96.4% in the real dataset, while 99.4% in the test set and maintained in the real dataset based on the Vessel-CTGAN-SVM. However, the recognition rates of the machine learning model based on the WoodFiber-CTGAN-BPNN was 76.6% in the test set and 75.7% in the real dataset, while 74.1% in the test set and 77.9% in the real dataset based on WoodFiber-CTGAN-SVM. These results indicated that both BPNN and SVM algorithms based on vessel geometry data could achieve high recognition levels, while the SVM algorithm performs better. However, machine learning models based on wood fiber geometry data could not achieve satisfactory recognition results. However, compared with the BPNN and SVM models without CTGAN-enhanced data, the recognition rates of the models have still improved. Compared with the Vessel-BPNN and Vessel-SVM models, the recognition rates have increased by 6.6 and 15.7 percentage points, respectively, after using the CTGAN data enhancement method. Compared with the WoodFiber-BPNN and WoodFiber-SVM models, the recognition rates have increased by 9.6 and 4.0 percentage points, respectively, after using the CTGAN data enhancement method.

**Table 5:** Recognition rates of models based on different cell geometry features and different machine algorithms.

Recognition algorithms	Recognition rate of test set	Recognition rate of real data
Vessel-BPNN	89.8%	
Vessel-SVM	83.7%	
WoodFiber-BPNN	65.9%	
WoodFiber-SVM	73.9%	
Vessel-CTGAN-BPNN	99.2%	96.4%
Vessel-CTGAN-SVM	99.4%	99.4%
WoodFiber-CTGAN-BPNN	76.6%	75.5%
WoodFiber-CTGAN-SVM	74.1%	77.9%

**Figure 6:**
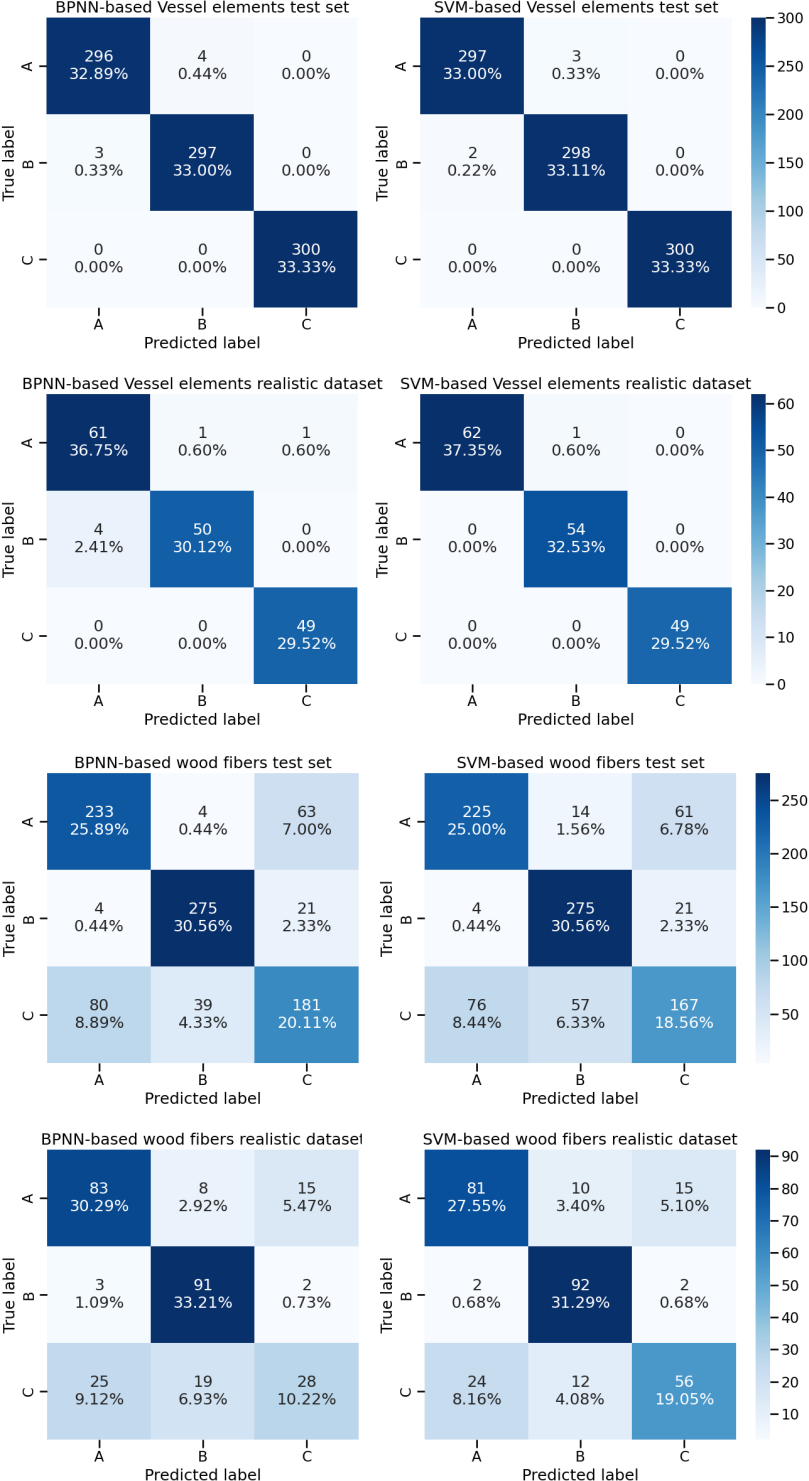
Identification results based on vessel cell and wood fiber cell geometry data for three tree species, Note: A, B and C in the diagram represent *Cyclobalanopsis chungii*, *Cyclobalanopsis gilva* and *Cyclobalanopsis glauca* respectively.

The confusion matrices based on the CTGAN simulated vessel and wood fiber cell geometry data were plotted (**Figure 6**). Two machine learning models, BPNN and SVM, were trained based on CTGAN simulated anatomical data of two types of cells. The BPNN model trained on the CTGAN simulated anatomical data of vessel cells achieved high accuracy in the test set, with only three prediction errors in the identification of 299 *Cyclobalanopsis chungii* tree species, four errors in the identification of301 *Cyclobalanopsis gilva* tree species and no errors in the identification of *Cyclobalanopsis glauca*. Similarly, the SVM model trained on the CTGAN simulated anatomical data of vessel cells also achieved high accuracy in the test set, with only 2 and 3 prediction errors in the identification of 299 *Cyclobalanopsis chungii* and 301 *Cyclobalanopsis gilva* tree species, respectively, and no errors in the identification of *Cyclobalanopsis glauca*. It is worth noting that Vessel-CTGAN-BPNN and Vessel-CTGAN-SVM also achieved high recognition accuracy on the real cell dataset. In the real *Cyclobalanopsis chungii* cell dataset, Vessel-CTGAN-BPNN misclassified only 4 out of 65 wood cell data; in the real *Cyclobalanopsisgilva* cell dataset, Vessel-CTGAN-BPNN misclassified only 1 out of 51 wood cell data, and in the real *Cyclobalanopsis glauca* cell dataset, Vessel-CTGAN-BPNN misclassified only 1 out of 50 wood cell data. Similarly, Vessel-CTGAN-SVM achieved 100% accuracy in the real *Cyclobalanopsis chungii* cell dataset, misclassified only 1 out of 55 wood cell data in the real *Cyclobalanopsis gilva* tree species cell dataset, and achieved 100% accuracy in the real *Cyclobalanopsis glauca* tree species cell dataset. Except for vessel cells, the performance of the SVM and BPNN models trained on CTGAN-simulated anatomical data of wood fiber cells is relatively worse. Nevertheless, the performance of these models is very close in both the test set and the real dataset, which is enough to illustrate that CTGAN-simulated anatomical data of cells can train good wood identification models for real datasets.

### 3.5 Model interpretability

#### 3.5.1 Selection of local data for LIME

A LIME linear model was established using Matlab software to fit the identification of BPNN and SVM models on specific data and to demonstrate how the two machine-learning models identify specific wood species. The LIME modeling was conducted on vessel and wood fiber cells of three *Cyclobalanopsis* species. Six samples of two types of cells from the three species were randomly selected for model interpretation and analysis, as presented in **Table 6**. Here, the 10th, 89th, and 150th vessel cell data and the 14th, 114th, and 230th wood fiber cell data were analyzed. Considering that recognition rate of model was unrelated to model interpretation, BPNN model was randomly selected for model interpretation based on vessel features and SVM model was chosen for model interpretation based on wood fiber features.

**Table 6:** Data on specific cell characteristics of three tree species used for LIME model interpretation.

	species	*Cyclobalanopsis gilva*	*Cyclobalanopsis chungii*	*Cyclobalanopsis glauca*
Vessel elements	Wall thickness/	11.5074	5.66473	0.05444
	Tangential diameter/	114.326	157.148	165.83
	Tangential diameter of lumina/	102.818	145.819	157.268
	ratio of wall to lumina	0.11192	0.0777	4.28088
	Area/µm^2^	10858.1	22712.4	22385.7
	Area of lumina/µm^2^	9306.97	19471.3	20260.5
	Substantial rate	0.14285	0.1427	0.09494
	Circumference/µm^2^	369.944	536.544	531.726
	Circumference of lumina/µm^2^	342.762	497.819	505.881
Wood fibers	Area/µm^2^	196.275	194.301	63.3392
	Area of lumina/µm^2^	59.5437	12.484	19.016
	Substantial rate	0.69663	0.93575	0.69977

Observing the vessel cell data interpreted by the LIME model through random selection, it can be seen that the difference in wall thickness feature is the greatest among the three tree species. Among other features, there are always two tree species with similar values. Theoretically, we hope that the LIME model’s explanation technique is in line with our prior knowledge and that tree species identification is based on the wall thickness feature. Observing the wood fiber cell data that the LIME model interprets through random selection, none of the three features make sufficient decisions. Theoretically, we hope that the LIME model’s explanation technique can integrate multiple types of information to make decisions.

#### 3.5.2 Model interpretation based on vessel features

In order to explore how the BPNN model identifies wood species based on the geometric features of vessel cells, a simple linear model would be used as a local model to fit the BPNN model. Specifically, we

Replaced the activation function of each hidden unit with a ReLU function to achieve this transformation. Then, the linear model’s output was calculated using the following formula(13).


(13) $$y=\sum_{i=1}^{n}w_{i}x_{i}+b$$

**Figure 7:**
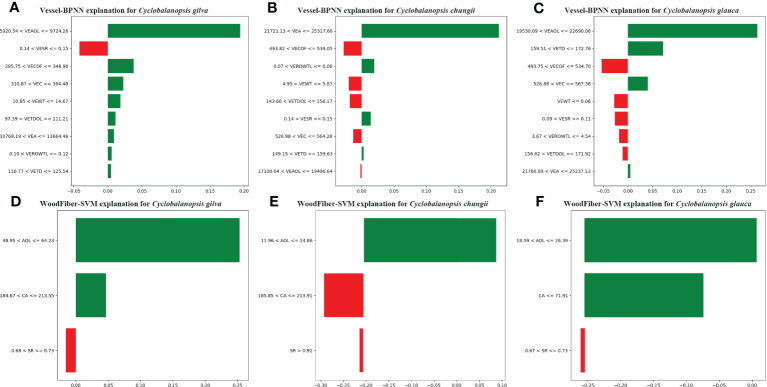
LIME Model Interpretation: How Machine Learning Identifies Tree Species Based on Geometric Features of Vessel Cells **(A–C)** and Wood Fiber Cells **(D–F)**, with the Following Letter Symbols Representing: Vessel Wall Thickness (VEWT), Vessel Tangential Diameter (VETD), Vessel Tangential Diameter of Lumina (VETDOL), Vessel Ratio of Wall to Lumina (VEROWTL), Vessel Area (VEA), Vessel Area of Lumina (VEAOL), Vessel Substantial Rate (VESR), Vessel Circumference (VEC), Vessel Circumference of Lumina (VECOF), Fiber Area (CA), Fiber Substantial Rate (SR), and Fiber Area of Lumina (AOL).

Among them, n was the number of features, $w_{i}$ was the weight of the i-th feature, $x_{i}$ was the value of the i-th feature, and b was the bias term. This relatively simple linear model had good interpretability, which could obtain the interpretation results of the model for predicting specific conduit data. As shown in **Figure 7**, the geometric features of the conduit cells had a specific impact on the model identification when the BPNN model identified 3 pieces of wood conduit data in **Table 6** (Swirszcz et al., 2009; Lozano et al., 2011).

The BPNN model relies mainly on the characteristics of vessel’s area of lumina (VEAOL) and vessel’s area(VEA) for identifying three types of *Cyclobalanopsis* wood species. VEAOL plays a vital role in identifying *Cyclobalanopsis gilva* and *Cyclobalanopsis glauca*, while VEA plays a significant role in identifying *Cyclobalanopsis chungii* (**Figure 7**). By analyzing the vessel’s substantial rate (VESR) feature values in combination with the prior knowledge from **Tables 3**, **6**, the average value of *Cyclobalanopsis gilva* is 0.118, and the sample value for model interpretation is 0.1428; the average value of *Cyclobalanopsis chungii* is 0.156, and the sample value for model interpretation is 0.1427; the average value of *Cyclobalanopsis glauca* is 0.138, and the sample value for model interpretation is 0.0949. From **Figure 7A**, the model considers that a value between 0.14 and 0.15 has a negative effect on identifying the cell data as *Cyclobalanopsis gilva*; from **Figure 7B**, the model considers that a value between 0.14 and 0.15 has a positive effect on identifying the cell data as *Cyclobalanopsis chungii*; from **Figure 7C**, the model considers that a value between 0.09 and 0.11 has a negative effect on identifying the cell data as *Cyclobalanopsis glauca*. Therefore, the model conforms well to our prior knowledge in judging the VESR feature point.

#### 3.5.3 Model interpretation based on wood fiber features

In order to explore how the SVM model identified tree species based on the geometric features of wood fiber cells, a simple linear model would be used as a local model to fit the SVM. For the SVM support vector machine model, the interpretation method of LIME technology was as follows: first, we calculated the weight of each support vector in a specific instance. It could be calculated by the following formula (14).


(14) $$w_{i}=\alpha_{i}y_{i}K\left(x_{i},x\right)$$

Among them, $\alpha_{i}$ was the Lagrange multiplier of the i-th support vector, $y_{i}$ was the class label corresponding to the i-th support vector, $K\left(x_{i},x\right)$ was the value of the kernel function, $x_{i}$ was the feature vector of the i-th support vector, and x was the feature vector of the specific instance.

Next, the weights of all support vectors were added up to obtain the explanation result for a specific instance (15).


(15) $$y=\sum_{i=1}^{m}w_{i}$$

Among them, m was the number of support vectors (Swirszcz et al., 2009; Lozano et al., 2011).

Using this method, It could be obtained the weight of each feature in the SVM model for a specific instance and how these weights contribute to the model’s prediction results (**Figure 7**).

The SVM model mainly relies on the features of fiber’s area of lumina (AOL) and fiber’s area (CA) to identify three types of *Cyclobalanopsis* wood species, with AOL having the most significant weight and CA coming second (**Figure 7**). Analyzing the AOL feature value more specifically, the SVM model believes that *Cyclobalanopsis gilva* should have the largest AOL, followed by *Cyclobalanopsis glauca*, and finally *Cyclobalanopsis chungii*. By observing **Table 2** and extracting prior knowledge, it can be seen that the average value of *Cyclobalanopsis gilva* is 89.580, *Cyclobalanopsis glauca* is 21.006, and *Cyclobalanopsis chungii* is 18.198. Therefore, the SVM model conforms to our prior knowledge in analyzing the AOL feature value.

## 4 Conclusion

In this study, two machine learning models, the BP neural network and SVM models, were established based on quantitative geometric characteristics data of vessels and wood fibers to identify the three specie s of *Cyclobalanopsis*, and explained those models with LIME-based model interpretation.

1. The machine learning model constructed based on the geometric characteristics data of vessel elements could effectively identify the three species. Additionally, SVM model was of higher prediction accuracy than BPNN model.
2. The CTGAN model could be effectively applied to enhance geometric characteristic dataset of wood species. The machine learning models trained on the dataset enhanced by the CTGAN model had a high recognition rate for the geometric characteristics of actual microscopic features.
3. The use of LIME model interpretation techniques can effectively verify whether the decision and analysis of the wood identification model conforms to human knowledge. In the field of wood science, model interpretation techniques should be supplemented and further discussed and researched as a model evaluation direction beyond the traditional recognition rate index.

The CTGAN and LIME technologies have been preliminarily verified in our work, but there are still some limitations. Firstly, this research work deserves a deeper replication on a larger microstructure data set of wood, which will further demonstrate the significance of our work on a larger scale. Secondly, the LIME model explanation technology can not only build modeling analysis based on numerical features such as micro features but also has a place in image analysis, although it is not perfect, it is worthy of exploration. Finally, LIME is just one of the many artificial intelligence model explanation technologies, and more model explanation technologies should be introduced to the tree species identification field to find the optimal explanation model (Lundberg and Lee, 2017; Shrikumar et al., 2017).

In terms of future research, we offer the following suggestions. Firstly, we recommend replicating and validating the methods on a larger data set. Secondly, models, including YOLO, SegNet, UNet, and the SAM model (Redmon et al., 2016; Badrinarayanan et al., 2017; Huang et al., 2020; Kirillov et al., 2023), which has recently gained popularity, have the potential for introducing wood cell structure analysis and feature extraction. This potential could lead to a significant decrease in the cost of manually collecting features (Yang et al., 2022a; Yang et al., 2022b). Lastly, to create true, industrialized, and intelligent forestry applications, it would be beneficial to further combine model interpretation technology with traditional wood anatomy and wood recognition models.

## Data availability statement

The raw data supporting the conclusions of this article will be made available by the authors, without undue reservation.

## Author contributions

XW and CL conducted the preparation of *Cyclobalanopsis* wood microsections and captured geometric features measurements. BC contributed to the construction of the BPNN model, while JL provided guidance on determining the geometric feature range of the wood used in this study. WZ provided the construction of the CTGAN model and SVM model, and conducted model explanations based on LIME for the machine learning models. WZ, BC, and ZY conducted data analysis and image rendering, and together wrote the manuscript, with XG providing guidance and oversight on the overall experimental process and manuscript revision. All authors provided operational feedback and worked together to improve the content of the paper. All authors contributed to the article and approved the submitted version.

## References

[B1] Agatonovic-Kustrin S. Beresford R. (2000). Basic concepts of artificial neural network (ann) modeling and its application in pharmaceutical research. J. Pharm. Biomed. Anal. 22, 717–727. doi: 10.1016/S0731-7085(99)00272-1 10815714

[B2] Alqarni A. A. El-Alfy E.-S. M. (2022). Improving intrusion detection for imbalanced network traffic using generative deep learning. Int. J. Adv. Comput. Sci. Appl. 13. doi: 10.14569/IJACSA.2022.01304109

[B3] Angyalossy V. Pace M. R. Evert R. F. Marcati C. R. Oskolski A. A. Terrazas T.. (2016). Iawa list of microscopic bark features. IAWA J. 37, 517–615. doi: 10.1163/22941932-20160151

[B4] Assefa S. A. Dervovic D. Mahfouz M. Tillman R. E. Reddy P. Veloso M. (2020). “Generating synthetic data in finance: opportunities, challenges and pitfalls,” in Proceedings of the First ACM International Conference on AI in Finance, Vol. 1–8.

[B5] Badrinarayanan V. Kendall A. Cipolla R. (2017). “Segnet: a deep convolutional encoder-decoder architecture for image segmentation,” in IEEE transactions on pattern analysis and machine intelligence, Vol. 39. 2481–2495. 10.1109/TPAMI.2016.2644615 28060704

[B6] Bergesen H. O. Parmann G. Thommessen Ø.B. (2018). “Convention on international trade in endangered species of wild fauna and flora (cites),” in Yearbook of International Cooperation on Environment and Development 1998–99, Routledge. 156–157.

[B7] Bourou S. El Saer A. Velivassaki T.-H. Voulkidis A. Zahariadis T. (2021). A review of tabular data synthesis using gans on an ids dataset. Information 12, 375. doi: 10.3390/info12090375

[B8] Carlquist S. (2013). Comparative wood anatomy: systematic, ecological, and evolutionary aspects of dicotyledon wood (Berlin, Germany: Springer Science & Business Media).

[B9] Chang Q. Chen Q. Wang X. (2005). “Scaling gaussian rbf kernel width to improve svm classification,” in 2005 international conference on neural networks and brain (IEEE), Vol. 1. 19–22.

[B10] Cifuentes A. Peña J-M Suarez F Larre O Ramırez D. (2023). A modified CTGAN-Plus-Features based method for optimal asset allocation. Tech. rep. arXiv.

[B11] Coday A E. Maun K. W. (1997). Identification of hardwoods: a microscope key (Bucknalls Lane, Watford, Herts: Constructions Research Communications Ltd).

[B12] Courville A. Bengio Y. (2014). Generative adversarial nets. Advanc Neural 63, 139–144. doi: 10.1145/3422622

[B13] DeOliveira J. Gerych W. Koshkarova A. Rundensteiner E. Agu E. (2022). “Har-ctgan: a mobile sensor data generation tool for human activity recognition,” in 2022 IEEE International Conference on Big Data (Big Data) (IEEE). 5233–5242.

[B14] Fang M. L. Dhami D. S. Kersting K. (2022). “Dp-ctgan: differentially private medical data generation using ctgans,” in Artificial Intelligence in Medicine: 20th International Conference on Artificial Intelligence in Medicine, AIME 2022, Halifax, NS, Canada, , June 14–17, 2022. 178–188.

[B15] Finkeldey R. Leinemann L. Gailing O. (2010). Molecular genetic tools to infer the origin of forest plants and wood. Appl. Microbiol. Biotechnol. 85, 1251–1258. doi: 10.1007/s00253-009-2328-6 19911178 PMC2807931

[B16] Friedrichs F. Igel C. (2005). Evolutionary tuning of multiple svm parameters. Neurocomputing 64, 107–117. doi: 10.1016/j.neucom.2004.11.022

[B17] Gasson P. Miller R. Stekel D. J. Whinder F. Ziemińska K. (2010). Wood identification of dalbergia nigra (cites appendix i) using quantitative wood anatomy, principal components analysis and naïve bayes classification. Ann. Bot. 105, 45–56. doi: 10.1093/aob/mcp270 19884155 PMC2794071

[B18] Gauch H. G. Hwang J. G. Fick G. W. (2003). Model evaluation by comparison of model-based predictions and measured values. Agron. J. 95, 1442–1446. doi: 10.2134/agronj2003.1442

[B19] Grabner M. Salaberger D. Okochi T. (2009). “The need of high resolution-x-ray ct in dendrochronology and in wood identification,” in 2009 Proceedings of 6th International Symposium on Image and Signal Processing and Analysis (IEEE). 349–352.

[B20] Hafemann L. G. Oliveira L. S. Cavalin P. (2014). “Forest species recognition using deep convolutional neural networks,” in 2014 22Nd international conference on pattern recognition (IEEE). 1103–1107.

[B21] Han G. Liu S. Chen K. Yu N. Feng Z. Song M. (2022). “Imbalanced sample generation and evaluation for power system transient stability using ctgan,” in Intelligent Computing & Optimization: Proceedings of the 4th International Conference on Intelligent Computing and Optimization 2021 (ICO2021) 3 (Springer). 555–565.

[B22] He T. Marco J. Soares R. Yin Y. Wiedenhoeft A. C. (2019). Machine learning models with quantitative wood anatomy data can discriminate between swietenia macrophylla and swietenia mahagoni. Forests 11, 36. doi: 10.3390/f11010036

[B23] Huang H. Lin L. Tong R. Hu H. Zhang Q. Iwamoto Y.. (2020). “Unet 3+: a full-scale connected unet for medical image segmentation,” in ICASSP 2020-2020 IEEE International Conference on Acoustics, Speech and Signal Processing (ICASSP) (IEEE). 1055–1059.

[B24] Hwang S. Kobayashi K. Sugiyama J. (2020). “Evaluation of a model using local features and a codebook for wood identification,” in In IOP Conference Series: Earth and Environmental Science (IOP Publishing), Vol. 415. 012029.

[B25] Hwang S.-W. Kobayashi K. Zhai S. Sugiyama J. (2018). Automated identification of lauraceae by scale-invariant feature transform. J. Wood Sci. 64, 69–77. doi: 10.1007/s10086-017-1680-x

[B26] Hwang S.-W. Sugiyama J. (2021). Computer vision-based wood identification and its expansion and contribution potentials in wood science: a review. Plant Methods 17, 1–21. doi: 10.1186/s13007-021-00746-1 33910606 PMC8082842

[B27] Ingre B. Yadav A. (2015). “Performance analysis of nsl-kdd dataset using ann,” in 2015 international conference on signal processing and communication engineering systems (IEEE). 92–96.

[B28] Jansen S. Kitin P. De Pauw H. Idris M. Beeckman H. Smets E. (1998). Preparation of wood specimens for transmitted light microscopy and scanning electron microscopy. Belgian J. Bot. 13 (1), 41–49.

[B29] Joachims T. (1998). Making large-scale SVM learning practical. Tech. rep. Tech. Rep.

[B30] Kirillov A. Mintun E. Ravi N. Mao H. Rolland C. Gustafson L.. (2023). Segment anything. arXiv preprint arXiv:2304.02643.

[B31] Kobayashi K. Hwang S.-W. Okochi T. Lee W.-H. Sugiyama J. (2019a). Non-destructive method for wood identification using conventional x-ray computed tomography data. J. Cultural Heritage 38, 88–93. doi: 10.1016/j.culher.2019.02.001

[B32] Kobayashi K. Kegasa T. Hwang S.-W. Sugiyama J. (2019b). Anatomical features of fagaceae wood statistically extracted by computer vision approaches: some relationships with evolution. PLoS One 14, e0220762. doi: 10.1371/journal.pone.0220762 31404108 PMC6690550

[B33] Koch G. Haag V. Heinz I. Richter H.-G. Schmitt U. (2015). Control of internationally traded timber-the role of macroscopic and microscopic wood identification against illegal logging. J. Forensic Res. 6, 1000317. doi: 10.4172/2157-7145.1000317

[B34] Kuroda K. (1987). Hardwood identificatlon using a microcomputer and iawa codes. IAWA J. 8, 69–77. doi: 10.1163/22941932-90001030

[B35] Kwon O. Lee H. G. Lee M.-R. Jang S. Yang S.-Y. Park S.-Y.. (2017). Automatic wood species identification of korean softwood based on convolutional neural networks. J. Korean Wood Sci. Technol. 45, 797–808. doi: 10.5658/WOOD.2017.45.6.797

[B36] Lee J. Lee O. (2021). Ctgan vs tgan? which one is more suitable for generating synthetic eeg data. J. Theor. Appl. Inf. Technol. 99.

[B37] Lens F. Liang C. Guo Y. Tang X. Jahanbanifard M. da Silva F. S. C.. (2020). Computer-assisted timber identification based on features extracted from microscopic wood sections. IAWA J. 41, 660–680. doi: 10.1163/22941932-bja10029

[B38] Lions A. (2011). Convention on international trade in endangered species of wild fauna and flora. 10.1159/000459796 712806

[B39] Liu S. He T. Wang J. Chen J. Guo J. Jiang X.. (2022). Can quantitative wood anatomy data coupled with machine learning analysis discriminate cites species from their look-alikes? Wood Sci. Technol. 56, 1567–1583. doi: 10.1007/s00226-022-01404-y

[B40] Lozano A. Swirszcz G. Abe N. (2011). “Group orthogonal matching pursuit for logistic regression,” in Proceedings of the fourteenth international conference on artificial intelligence and statistics (JMLR Workshop and Conference Proceedings). 452–460.

[B41] Lundberg S. M. Lee S.-I. (2017). A unified approach to interpreting model predictions. Adv. Neural Inf. Process. Syst. 30.

[B42] Maas A. L. Hannun A. Y. Ng A. Y. (2013). “Rectifier nonlinearities improve neural network acoustic models,” in Proc. icml, Atlanta, Georgia, USA, Vol. 30. 3.

[B43] Mai C. Schmitt U. Niemz P. (2022). A brief overview on the development of wood research. Holzforschung 76, 102–119. doi: 10.1515/hf-2021-0155

[B44] Martins J. Oliveira L. Nisgoski S. Sabourin R. (2013). A database for automatic classification of forest species. Mach. Vision Appl. 24, 567–578. doi: 10.1007/s00138-012-0417-5

[B45] McAvaney B. J. Covey C. Joussaume S. Kattsov V. Kitoh A. Ogana W.. (2001). “Model evaluation,” in Climate change 2001: the scientific basis. contribution of WG1 to the third assessment report of the IPCC (TAR) (Cambridge: Cambridge University Press), 471–523.

[B46] Milli S. Schmidt L. Dragan A. D. Hardt M. (2019). “Model reconstruction from model explanations,” in Proceedings of the Conference on Fairness, Accountability, and Transparency. 1–9.

[B47] Mirza M. Osindero S. (2014). Conditional generative adversarial nets. arXiv preprint arXiv. doi: 10.48550/arXiv.1411.1784

[B48] Mishra S. Sturm B. L. Dixon S. (2017). Local interpretable model-agnostic explanations for music content analysis. ISMIR 53, 537–543.

[B49] Mohan S. Venkatachalapathy K. Sudhakar P. (2014). An intelligent recognition system for identification of wood species. J. Comput. Sci. 10, 1231. doi: 10.3844/jcssp.2014.1231.1237

[B50] Ohyama M. Baba K. Itoh T. (2001). Wood identification of japanese cyclobalanopsis species (fagaceae) based on dna polymorphism of the intergenic spacer between trn t and trn l 5’ exon. J. Wood Sci. 47, 81–86. doi: 10.1007/BF00780554

[B51] Peltola T. (2018). Local interpretable model-agnostic explanations of bayesian predictive models *via* kullback-leibler projections. arXiv preprint arXiv.

[B52] Peña J.-M. Suárez F. Larré O. Ramírez D. Cifuentes A. (2023). A modified ctgan-plus-features based method for optimal asset allocation. arXiv preprint arXiv.

[B53] Peng B. Zhang A. Zhang T. (2021). Credit scoring model in imbalanced data based on cnn-atcn. Preprint from Research Square, 04 Aug 2021. doi: 10.21203/rs.3.rs-714980/v1

[B54] Poursabzi-Sangdeh F. Goldstein D. G. Hofman J. M. Wortman Vaughan J. W. Wallach H. (2021). “Manipulating and measuring model interpretability,” in Proceedings of the 2021 CHI conference on human factors in computing systems. 1–52.

[B55] Raschka S. (2018). Model evaluation, model selection, and algorithm selection in machine learning. arXiv preprint arXiv.

[B56] Ravindran P. Costa A. Soares R. Wiedenhoeft A. C. (2018). Classification of cites-listed and other neotropical meliaceae wood images using convolutional neural networks. Plant Methods 14, 1–10. doi: 10.1186/s13007-018-0292-9 29588649 PMC5865295

[B57] Ravindran P. Wiedenhoeft A. C. (2020). Comparison of two forensic wood identification technologies for ten meliaceae woods: computer vision versus mass spectrometry. Wood Sci. Technol. 54, 1139–1150. doi: 10.1007/s00226-020-01178-1

[B58] Redmon J. Divvala S. Girshick R. Farhadi A. (2016). “You only look once: unified, real-time object detection,” in Proceedings of the IEEE conference on computer vision and pattern recognition. 779–788.

[B59] Ribeiro M. T. Singh S. Guestrin C. (2016). “" why should i trust you?" explaining the predictions of any classifier,” in Proceedings of the 22nd ACM SIGKDD international conference on knowledge discovery and data mining. 1135–1144.

[B60] Richter H. G. Grosser D. Heinz I. Gasson P. E. (2004). Iawa list of microscopic features for softwood identification. Iawa J. 25, 1–70. doi: 10.1163/22941932-90000349

[B61] Romagnolj M. Sarlatto M. Terranova F. Bizzarrj E. Cesettj S. (2007). Wood identification in the cappella palatina ceiling (12th century) in palermo (sicily, italy). Iawa J. 28, 109–124. doi: 10.1163/22941932-90001628

[B62] Rosa da Silva N. De Ridder M. Baetens J. M. Van den Bulcke J. Rousseau M. Martinez Bruno O.. (2017). Automated classification of wood transverse cross-section micro-imagery from 77 commercial central-african timber species. Ann. For. Sci. 74, 1–14. doi: 10.1007/s13595-017-0619-0

[B63] Ruder S. (2016). An overview of gradient descent optimization algorithms. arXiv preprint arXiv.

[B64] Rumelhart D. E. Hinton G. E. Williams R. J. (1986). Learning representations by back-propagating errors. nature 323, 533–536. doi: 10.1038/323533a0

[B65] Sharma V. Yadav J. Kumar R. Tesarova D. Ekielski A. Mishra P. K. (2020). On the rapid and non-destructive approach for wood identification using atr-ftir spectroscopy and chemometric methods. Vibrational Spectrosc. 110, 103097. doi: 10.1016/j.vibspec.2020.103097

[B66] Shrikumar A. Greenside P. Kundaje A. (2017). “Learning important features through propagating activation differences,” in International conference on machine learning (PMLR). 3145–3153.

[B67] Sugiarto B. Prakasa E. Wardoyo R. Damayanti R. Dewi L. M. Pardede H. F.. (2017). “Wood identification based on histogram of oriented gradient (hog) feature and support vector machine (svm) classifier,” in 2017 2nd International conferences on Information Technology, Information Systems and Electrical Engineering (ICITISEE) (IEEE). 337–341.

[B68] Sun Y. Lin Q. He X. Zhao Y. Dai F. Qiu J.. (2021). Wood species recognition with small data: a deep learning approach. Int. J. Comput. Intell. Syst. 14, 1451–1460. doi: 10.2991/ijcis.d.210423.001

[B69] Swirszcz G. Abe N. Lozano A. C. (2009). Grouped orthogonal matching pursuit for variable selection and prediction. Adv. Neural Inf. Process. Syst. 22.

[B70] Torfi A. Fox E. A. Reddy C. K. (2022). Differentially private synthetic medical data generation using convolutional gans. Inf. Sci. 586, 485–500. doi: 10.1016/j.ins.2021.12.018

[B71] Tou J. Y. Lau P. Y. Tay Y. H. (2007). “Computer vision-based wood recognition system,” in Proceedings of International workshop on advanced image technology. 197–202.

[B72] Vellido A. Martín-Guerrero J. D. Lisboa P. J. (2012). “Making machine learning models interpretable,” in ESANN (Citeseer), Vol. 12. 163–172.

[B73] von Arx G. Carrer M. Crivellaro A. De Micco V. Fonti P. Lens F.. (2021). Q-net–a new scholarly network on quantitative wood anatomy. Dendrochronologia 70, 125890.

[B74] Von Arx G. Crivellaro A. Prendin A. L. Čufar K. Carrer M. (2016). Quantitative wood anatomy–practical guidelines. Front. Plant Sci. 7, 781. 27375641 10.3389/fpls.2016.00781 PMC4891576

[B75] Wheeler E. A. (2011). Inside wood–a web resource for hardwood anatomy. Iawa J. 32, 199–211. doi: 10.1163/22941932-90000051

[B76] Wheeler E. A. Baas P. (1998). Wood identification-a review. IAWA J. 19, 241–264. doi: 10.1163/22941932-90001528

[B77] Wheeler E. A. Baas P. Gasson P. E. (1989). Iawa list of microscopic features for hardwood identification. IAWA Journal (International Association of Wood Anatomists) 10 (3), 219–332.

[B78] Wiedenhoeft A. C. Simeone J. Smith A. Parker-Forney M. Soares R. Fishman A. (2019). Fraud and misrepresentation in retail forest products exceeds us forensic wood science capacity. PloS One 14, e0219917. doi: 10.1371/journal.pone.0219917 31344141 PMC6657862

[B79] Xiao Y. Wang H. Xu W. (2014). Parameter selection of gaussian kernel for one-class svm. IEEE Trans. cybernetics 45, 941–953. doi: 10.1109/TCYB.2014.2340433 25099969

[B80] Xu L. Skoularidou M. Cuesta-Infante A. Veeramachaneni K. (2019). Modeling tabular data using conditional gan. Adv. Neural Inf. Process. Syst. 32.

[B81] Yadav A. R. Anand R. S. Dewal M. Gupta S. (2014). “Analysis and classification of hardwood species based on coiflet dwt feature extraction and weka workbench,” in 2014 International Conference on Signal Processing and Integrated Networks (SPIN) (IEEE). 9–13.

[B82] Yadav A. R. Anand R. S. Dewal M. Gupta S. (2015a). Hardwood species classification with dwt based hybrid texture feature extraction techniques. Sadhana 40, 2287–2312. doi: 10.1007/s12046-015-0441-z

[B83] Yadav A. R. Anand R. S. Dewal M. Gupta S. (2015b). Multiresolution local binary pattern variants based texture feature extraction techniques for efficient classification of microscopic images of hardwood species. Appl. Soft Computing 32, 101–112. doi: 10.1016/j.asoc.2015.03.039

[B84] Yadav A. R. Dewal M. Anand R. S. Gupta S. (2013). “Classification of hardwood species using ann classifier,” in 2013 Fourth national conference on computer vision, pattern recognition, image processing and graphics (NCVPRIPG) (IEEE). 1–5.

[B85] Yang Z. Li L. Luo W. (2022a). Pdnet: improved yolov5 nondeformable disease detection network for asphalt pavement. Comput. Intell. Neurosci. 2022. doi: 10.1155/2022/5133543 PMC9283017 35845879

[B86] Yang Z. Ni C. Li L. Luo W. Qin Y. (2022b). Three-stage pavement crack localization and segmentation algorithm based on digital image processing and deep learning techniques. Sensors 22, 8459. doi: 10.3390/s22218459 36366156 PMC9656577

[B87] Yuliastuti E. Suprijanto Sasi S. R. (2013). “Compact computer vision system for tropical wood species recognition based on pores and concentric curve,” in 2013 3rd International Conference on Instrumentation Control and Automation (ICA) (IEEE). 198–202.

[B88] Zhang J. Nieminen K. Serra J. A. A. Helariutta Y. (2014). The formation of wood and its control. Curr. Opin. Plant Biol. 17, 56–63. doi: 10.1016/j.pbi.2013.11.003 24507495

[B89] Zhang Q.-s. Zhu S.-C. (2018). Visual interpretability for deep learning: a survey. Front. Inf. Technol. Electronic Eng. 19, 27–39. doi: 10.1631/FITEE.1700808

